# The changing food outlet distributions and local contextual factors in the United States

**DOI:** 10.1186/1471-2458-14-42

**Published:** 2014-01-16

**Authors:** Hsin-Jen Chen, Youfa Wang

**Affiliations:** 1 Johns Hopkins Global Center on Childhood Obesity, Department of International Health, Human Nutrition Program, Bloomberg School of Public Health, Johns Hopkins University, Baltimore, MD, USA; 2 Department of Social and Preventive Medicine, School of Public Health and Health Professions, University at Buffalo, State University of New York, Buffalo, NY, USA

## Abstract

**Background:**

Little is known about the dynamics of the food outlet distributions associated with local contextual factors in the U.S. This study examines the changes in food stores/services at the 5-digit Zip Code Tabulated Area (ZCTA5) level in the U.S., and assesses contextual factors associated with the changes.

**Methods:**

Data from 27,878 ZCTA5s in the contiguous United States without an extreme change in the number of 6 types of food stores/services (supermarkets, small-size grocery stores, convenience stores, fresh/specialty food markets, carry-out restaurants, and full-service restaurants) were used. ZCTA5s’ contextual factors were from the 2000 Census. Numbers of food stores/services were derived from the Census Business Pattern databases. Linear regression models assessed contextual factors’ influences (racial/ethnic compositions, poverty rate, urbanization level, and foreign-born population%) on 1-year changes in food stores/services during 2000–2001, adjusted for population size, total business change, and census regions.

**Results:**

Small-size grocery stores and fresh/specialty food markets increased more and convenience stores decreased more in Hispanic-predominant than other areas. Among supermarket-free places, new supermarkets were less likely to be introduced into black-predominant than white-predominant areas (odds ratio (OR) = 0.52, 95% CI = 0.30-0.92). However, among areas without the following type of store at baseline, supermarket (OR = 0.48 (0.33-0.70)), small-size grocery stores (OR = 1.32 (1.08-1.62)), and fresh/specialty food markets (OR = 0.70 (0.53-0.92)) were less likely to be introduced into areas of low foreign-born population than into areas of high foreign-born population. Higher poverty rate was associated with a greater decrease in supermarket, a less decrease in small-size grocery stores, and a less increase in carry-out restaurants (all p for trends <0.001). Urban areas experienced more increases in full-service and carry-out restaurants than suburban areas.

**Conclusions:**

Local area characteristics affect 1-year changes in food environment in the U.S. Hispanic population was associated with more food stores/services capable of supplying fresh food items. Black-predominant and poverty-afflicted areas had a greater decrease in supermarkets. Full-service and carry-out restaurants increased more in urban than suburban areas. Foreign-born population density was associated with introduction of grocery stores and fresh/specialty food markets into the areas.

## Background

In contemporary society of the United States, food stores and services are channels for people to fulfill their energy requirements and their desires of foods. Stores and services in local communities may impose obesity risks because accessibility to these stores can influence residents’ eating patterns [1,2]. A growing number of studies have shown that people living in places with lower accessibility or availability of healthy food options eat fewer fruits and vegetables, while the availability of fast food stores is associated with a more unhealthy dietary quality and with obesity [3-7]. To fight the obesity epidemic in the U.S., the local food landscape is an important target for population-based interventions [8,9].

In the U.S., poorer places and neighborhoods with a high concentration of African Americans often have fewer grocery stores or supermarkets, but have more carry-out and fast food restaurants [10,11]. Supermarkets or large size grocery stores are more likely to provide wholesome food choices, while carry-out and fast food restaurants often provide ready-to-go foods prepared using efficient procedures but with less healthy ingredients [11-18]. These neighborhoods are more likely to be “food deserts,” lacking availability or accessibility to healthy food options [19]. The cross-sectional picture of the correlation between local socioeconomic conditions and the built food environment, however, cannot reveal the dynamics of food outlet distribution and the neighborhood characteristics.

Changes in the quantities of food outlets reflect the changing demand and supply of the stores/services in the local market. Studying the factors affecting local food landscape can help us understand more about the “desertization” of built food environment. A study based on an urban borough in the U.S. found a greater stability of supermarket maintenance in wealthier and white-predominant neighborhoods compared to poorer neighborhoods and neighborhoods with other racial composition [20]. In order to cover the heterogeneous places in the U.S. and to better understand the question, we used U.S. national data to study the relationship between the changes in food outlet distribution and local racial/ethnic compositions, local foreign-born population, urbanization level, and poverty rate.

## Methods

### Database

The U.S. Census 2000 provided data on contextual factors such as urbanization level, racial/ethnic composition and poverty, while the Zip-code Business Pattern (ZBP) data provided information on numbers of food stores/services at the Zip code level. Given that boundaries of postal Zip codes and the local population composition could change over time, we used a 1-year short time frame after the year of 2000 Census to minimize the potential impact that may be caused by the long-term dynamics of population and Zip code re-designation.

### Census data 2000

The 5-digit Zip Code Tabulation Areas (ZCTA5s) in the contiguous United States were included. The unit for analysis in this study was the ZCTA5, which was designated to coincide with the 5-digit postal Zip code area of the year 2000 [21]. Locations with <300 residents were excluded from analysis. Places that showed a large increase or decrease in the number (>10) of stores of interest were excluded based on the extreme distributions. The final size for analysis was 27,878 geographic units. Median (lower-upper quartiles) of population and land area of these ZCTA5s was 3685 (1224–13828) residents and 41.4 (13.5-94.3) square miles.

### Census Zip-code business patterns 2000 and 2001

The Census County Business Patterns database annually releases information about local business establishments in the U.S. [22]. It is collected based on the Business Register of the Census Bureau, a comprehensive database of business and companies in the U.S. The postal Zip code level is the smallest scale of the data. Business establishments were categorized using the North America Industry Classification System (NAICS) codes. The data provided information about the numbers of each type of establishment opened in every Zip code and the estimated numbers of employees for each type of establishment.

### Outcome variables—food outlet at Zip code level and the changes in their numbers

In ZBP data of 2000 and 2001, we used the NAICS codes to identify six types of retail food stores and food services. All types of supermarkets and grocery stores had the same NAICS code (“445110”). We defined supermarkets as grocery stores ≥50 employees, since supermarkets had many more employees, i.e., about 7 times more than smaller grocery stores [11,23]. Small-size grocery stores of <10 employees were defined as small-size food providers. Convenience stores (NASIC = ”445120”) and those associated with gasoline stations (NAICS = ”447110”) were combined as convenience stores. Fresh/specialty food markets included those selling especially meat, fish/seafood and fruits/vegetables (NAICS = ”445210,” “445220,” “445230”). Full-service restaurants’ NAICS code was“722110.” Carry-out restaurants were defined as limited-service restaurants (“722211,” including fast-food restaurants), cafeterias (“722212”) and mobile food services (“722330”). The numbers of these six types of food stores/services were extracted from the ZBP data, and the changes in numbers from year 2000 to 2001 were calculated.

### Explanatory variables—area socio-demographic characteristics

Four major contextual variables of interest at the ZCTA5 level were obtained based on the 2000 Census data: local race/ethnicity composition, proportion of foreign-born population, urbanization level, and local poverty rate. These four factors were chosen to indicate local race/ethnicity-related culture and demand, economic development, and income, respectively.

The ZCTA5s were categorized into four mutually exclusive race/ethnicity composition groups: white-predominant (>50% of local residents self-identified as non-Hispanic white), black-predominant (>50% non-Hispanic black residents), Hispanic-predominant (>50% Hispanic residents) and racially-mixed (the rest) areas. A ZCTA5 was categorized as urban, suburban or rural if the majority of the residents in the area were living in urban-nucleus (urban centers with population ≥50000 and population density ≥1000 people per square mile), urban-cluster (places with population ≥2500 and <50000) or rural block groups, respectively. Tertiles were used to categorize foreign-born population proportion (0%-0.99%, 1.00% – 3.65%, >3.65%) and poverty rate (0% – 7.66%, 7.66%-14.18%, >14.18%) to create subgroups of even sample size.

### Covariates

Number of food stores/services is majorly determined by the local population/market size [17], thus we controlled for population size and total number of establishments in 2000 when examined how the residual differences in the outcomes were explained by the demographic and socioeconomic contexts of interest. Likewise, since the number of food stores/services would change with local economic/business dynamics, we additionally controlled for the difference in the numbers of the total number of establishments from 2000 to 2001. We grouped the ZCTA5s into 9 census regions/divisions, according to the U.S. Census Bureau: New England, Mid-Atlantic, South Atlantic, East South Central, West South Central, East North Central, West North Central, Mountain, and Pacific.

### Statistical analysis

Distributions of local demographics, socioeconomic conditions and food stores/services were explored and stratified by race/ethnicity composition. The distributions of changed numbers in stores/services among these four types of communities were calculated. One-way ANOVA was used to test for differences among the four types of areas.

The changes in the number of these food outlets were symmetrically distributed at the means around 0, with large standard deviations. Ordinary linear regression models were fit to test the relationships between changed number of food stores/services and all four contextual variables of interest, adjusting for population size, census region, total number of establishments, and the change in total number of establishments from 2000 to 2001. The six types of food stores/services were modeled by separate regressions. The least-square (adjusted) means of changed numbers in those stores/services among the four race/ethnicity composition areas were estimated as covariates were hold at their average levels. To test the trend across tertiles of poverty rate and foreign-born population, we used another set of models treating the tertiles as an ordinal variable.

These analyses were repeated among ZCTA5s where the focal food outlets were present at baseline year. On the other hand, since the number of food outlets could not decrease if the areas did not have the type of food outlet at baseline, we created binary variables indicating the introduction of the food outlet into areas without the store/service at baseline. Then, we applied logistic regression models to examine the odds ratios of the store/service being introduced into the area by different contextual conditions. Data management and analysis were performed using SAS 9.2 (SAS Institute, Cary NC).

## Results

### Characteristics of the four race/ethnicity predominant areas

The majority (88.7%) of the ZCTA5s were categorized as white-predominant areas, which were socioeconomically better off than the other three groups. (Table 1) They had the highest median income and number of high school graduates, and the lowest poverty and unemployment rates. Moreover, residents of this group of areas were less likely to be living in urban settings. Although white-predominant areas were the richest, population and business sizes were the smallest among the four types of areas in 2000. The mixed racial/ethnic and Hispanic-predominant areas had larger business sizes, were more urbanized and populous, and had more food stores/services than the white-predominant areas.

**Table 1:** The distribution of baseline sociodemographic characteristics and food outlets by local racial/ethnic composition: the ZCTA5-level analysis in the U.S. (N = 27878)

	**White-predominant**		**Black-predominant**		**Hispanic-predominant**		**Mixed racial/ethnic areas**	
	**areas**		**areas**		**areas**			
	**n = 24719**		**n = 1185**		**n = 779**		**n = 1195**	
	**Mean**	**SE**	**Mean**	**SE**	**Mean**	**SE**	**Mean**	**SE**
Population per ZIP code (person)	8644	(73)	15421	(489)	23586	(866)	21468	(592)
Population density (person/mi2)	0.80	(0.02)	3.53	(0.24)	5.44	(0.43)	4.75	(0.24)
Average race/ethnicity proportion								
White	88.45	(0.08)	24.65	(0.42)	20.05	(0.46)	33.49	(0.44)
Black	4.37	(0.05)	69.21	(0.41)	5.05	(0.31)	19.06	(0.51)
Hispanic	3.92	(0.04)	3.80	(0.19)	70.96	(0.51)	21.23	(0.49)
Asian	1.11	(0.02)	0.75	(0.04)	2.08	(0.14)	6.98	(0.33)
Average proportion foreign born population	3.77	(0.03)	4.28	(0.22)	28.46	(0.63)	17.34	(0.45)
Median income (K)	41.56	(0.10)	27.13	(0.29)	30.10	(0.33)	35.63	(0.42)
Poverty rate	11.12	(0.05)	27.01	(0.33)	25.78	(0.37)	22.00	(0.38)
Unemployment rate	5.14	(0.02)	11.71	(0.20)	11.34	(0.22)	10.51	(0.23)
Urbanization level (distribution of three categories) (%)								
Urban	28%	(0.3%)	53%	(1.4%)	57%	(1.8%)	62%	(1.4%)
Suburban	11%	(0.2%)	9%	(0.8%)	16%	(1.3%)	8%	(0.8%)
Rural	61%	(0.3%)	38%	(1.4%)	27%	(1.6%)	29%	(1.3%)
**Average number (per ZCTA5) of stores/business in 2000**								
Total business	220.70	(2.31)	262.13	(9.46)	394.65	(17.10)	461.28	(15.65)
Total grocery stores	1.96	(0.02)	4.71	(0.17)	6.36	(0.29)	5.54	(0.19)
Supermarket	0.59	(0.01)	0.63	(0.03)	1.00	(0.05)	1.08	(0.04)
Small size grocery stores	0.85	(0.01)	3.24	(0.14)	4.08	(0.23)	3.45	(0.15)
Fresh/specialty food stores	0.32	(0.01)	0.77	(0.05)	1.16	(0.08)	1.00	(0.06)
Convenience stores	3.50	(0.03)	5.56	(0.19)	6.36	(0.25)	6.23	(0.20)
Full-service restaurants	6.15	(0.07)	5.51	(0.24)	10.47	(0.46)	12.87	(0.49)
Carry-out food stores	6.59	(0.07)	9.04	(0.34)	13.81	(0.57)	15.76	(0.52)
**Average changed number (per ZCTA5) of stores during 2000 and 2001**								
Total business	+0.65	(0.08)	-3.06	(0.42)	+0.21	(0.57)	-0.75	(0.61)
Total grocery stores	-0.01	(0.004)	-0.05	(0.03)	+0.13	(0.05)	+0.04	(0.04)
Supermarkets	-0.01	(0.002)	-0.04	(0.01)	-0.03	(0.02)	-0.01	(0.01)
Small size grocery stores	+0.01	(0.004)	+0.01	(0.03)	+0.19	(0.05)	+0.02	(0.04)
Fresh/specialty food markets	+0.01	(0.002)	-0.01	(0.01)	+0.05	(0.03)	+0.02	(0.02)
Convenience stores	+0.06	(0.01)	+0.02	(0.04)	-0.13	(0.05)	+0.01	(0.04)
Full-service restaurants	+0.00	(0.01)	-0.07	(0.04)	+0.00	(0.07)	+0.11	(0.06)
Carry-out food stores	+0.11	(0.01)	+0.07	(0.05)	+0.21	(0.07)	+0.28	(0.07)

*ANOVA test p = 0.0584; p values for all the other variables are <0.05.

Supermarkets are the grocery stores of > =50 employees, while small grocery stores are those of <10 employees.

ZCTA: Zip-code Tabulation Area.

From 2000 to 2001, the total number of businesses decreased drastically in black-predominant areas, but small-size grocery and convenience stores and carry-out restaurants increased in number. Supermarkets decreased in all four types of racial/ethnic areas, while carry-out restaurants and grocery/smaller food stores increased universally. (Table 1) Nevertheless, the numbers of stores that more likely provided wholesome foods, i.e. fresh food markets and small-size grocery stores, increased more in Hispanic-predominant and racial-mixed areas than the other areas.

### Overall changes in food outlets by local characteristics

As shown in Table 2, the changes in the numbers of food stores/services varied with different local contextual variables after controlling for covariates. The adjusted changes in numbers of these stores/services remained significant among the four racial/ethnic groups of areas. Comparing Hispanic-predominant to other racial/ethnic compositions, small-size grocery stores and specialty fresh food markets increased significantly, while convenience stores decreased the most. Communities with a larger foreign-born population had a greater decrease in supermarkets (p for trend = 0.049). An area’s poverty rate restrained the increase of supermarkets and carry-out restaurants. Nevertheless, small-size grocery stores increased more in areas of middle or upper poverty rate tertiles by ~5 stores per 100 ZCTA5 areas. Full-service and carry-out restaurants increased more in urban than in suburban areas. (Table 2) Both types of restaurants increased significantly, about 12 more in per 100 areas in urban than in suburban ZCTA5 areas (p < 0.001 for the differences in changes in both types of restaurants). Moreover, the number of carry-out restaurants persistently grew significantly, regardless of contextual factors.

**Table 2:** The adjusted (least-square) mean changes of the food outlets across local contextual factors in the U.S. from 2000 to 2001

		**ΔSupermarkets**				**ΔSmall grocery stores**				**ΔConvenience stores**			
		**Expected change**^**a**^	**95% CI**			**Expected change**^**a**^	**95% CI**			**Expected change**^**a**^	**95% CI**		
**Race composition**													
	White-predominant	- 0.010	(- 0.016 ,	- 0.004)		+0.001	(- 0.012 ,	+0.014)	^H^	+0.070	(+0.054 ,	+0.087)	^H^
	Black-predominant	- 0.012	(- 0.033 ,	+0.008)		+0.019	(- 0.028 ,	+0.067)	^H^	+0.026	(- 0.034 ,	+0.086)	^H^
	Hispanic-predominant	- 0.026	(- 0.051 ,	- 0.001)		+0.160	(+0.102 ,	+0.218)	^W,B,M^	- 0.160	(- 0.234 ,	- 0.086)	^W,B,M^
	Mixed	+0.004	(- 0.017 ,	+0.024)		- 0.020	(- 0.067 ,	+0.026)	^H^	- 0.004	(- 0.064 ,	+0.055)	^H^
**Urbanization level**													
	Rural	- 0.010	(- 0.022 ,	+0.002)		+0.048	(+0.020 ,	+0.075)		- 0.044	(- 0.078 ,	- 0.009)	
	Suburban	- 0.016	(- 0.031 ,	+0.000)		+0.027	(- 0.009 ,	+0.063)		+0.009	(- 0.037 ,	+0.055)	
	Urban	- 0.008	(- 0.021 ,	+0.005)		+0.046	(+0.016 ,	+0.076)		- 0.016	(- 0.054 ,	+0.022)	
**Foreign born population% tertiles**													
	1 (Bottom tertile)	- 0.008	(- 0.021 ,	+0.005)		+0.037	(+0.006 ,	+0.067)		- 0.037	(- 0.075 ,	+0.002)	
	2 (Middle tertile)	- 0.014	(- 0.026 ,	- 0.001)		+0.040	(+0.012 ,	+0.069)		- 0.014	(- 0.050 ,	+0.022)	
	3 (Top tertile)	- 0.012	(- 0.024 ,	+0.000)		+0.043	(+0.016 ,	+0.071)		- 0.000	(- 0.036 ,	+0.035)	
		*p*_*trend*_*= .049*				*p*_*trend*_*= .678*				*p*_*trend*_*= .064*			
**Poverty rate% tertiles**													
	1 (Bottom tertile)	+0.001	(- 0.013 ,	+0.014)	^2, 3^	+0.029	(- 0.001 ,	+0.060)		- 0.015	(- 0.054 ,	+0.024)	
	2 (Middle tertile)	- 0.016	(- 0.029 ,	- 0.003)	^1^	+0.045	(+0.016 ,	+0.074)		- 0.008	(- 0.045 ,	+0.028)	
	3 (Top tertile)	- 0.018	(- 0.029 ,	- 0.008)	^1^	+0.046	(+0.022 ,	+0.070)		- 0.028	(- 0.058 ,	+0.003)	
		*p*_*trend*_*= .002*				*p*_*trend*_*= .199*				*p*_*trend*_*= .461*			
		**ΔFresh/specialty food markets**				**ΔFull-service restaurants**				**ΔCarry-out restaurants**			
		Expected change^a^	95% CI			Expected change^a^	95% CI			Expected change^a^	95% CI		
**Race composition**													
	White-predominant	+0.010	(+0.003 ,	+0.016)		- 0.018	(- 0.043 ,	+0.007)		+0.096	(+0.070 ,	+0.123)	
	Black-predominant	- 0.011	(- 0.034 ,	+0.012)	^H^	- 0.050	(- 0.140 ,	+0.039)		+0.155	(+0.062 ,	+0.249)	
	Hispanic-predominant	+0.050	(+0.022 ,	+0.078)	^B^	- 0.044	(- 0.154 ,	+0.067)		+0.133	(+0.018 ,	+0.248)	
	Mixed	+0.013	(- 0.009 ,	+0.036)		+0.069	(- 0.020 ,	+0.157)		+0.177	(+0.085 ,	+0.270)	
**Urbanization level**													
	Rural	+0.016	(+0.002 ,	+0.029)		+0.007	(- 0.044 ,	+0.059)		+0.158	(+0.104 ,	+0.211)	
	Suburban	+0.014	(- 0.004 ,	+0.032)		- 0.078	(- 0.147 ,	- 0.009)	^U^	+0.075	(+0.003 ,	+0.146)	^U^
	Urban	+0.017	(+0.002 ,	+0.031)		+0.038	(- 0.019 ,	+0.095)	^S^	+0.190	(+0.130 ,	+0.249)	^S^
**Foreign born population% tertiles**													
	1 (Bottom tertile)	+0.016	(+0.001 ,	+0.031)		- 0.017	(- 0.075 ,	+0.041)		+0.151	(+0.091 ,	+0.212)	
	2 (Middle tertile)	+0.014	(+0.000 ,	+0.028)		- 0.026	(- 0.080 ,	+0.029)		+0.123	(+0.067 ,	+0.180)	
	3 (Top tertile)	+0.016	(+0.003 ,	+0.030)		+0.010	(- 0.043 ,	+0.062)		+0.147	(+0.092 ,	+0.202)	
		*p*_*trend*_*= .988*				*p*_*trend*_*= .435*				*p*_*trend*_*= .802*			
**Poverty rate% tertiles**													
	1 (Bottom tertile)	+0.018	(+0.003 ,	+0.033)		+0.002	(- 0.057 ,	+0.060)		+0.211	(+0.150 ,	+0.272)	^2,3^
	2 (Middle tertile)	+0.013	(- 0.001 ,	+0.027)		- 0.016	(- 0.071 ,	+0.039)		+0.132	(+0.075 ,	+0.190)	^1^
	3 (Top tertile)	+0.015	(+0.004 ,	+0.027)		- 0.019	(- 0.064 ,	+0.027)		+0.078	(+0.031 ,	+0.126)	^1^
		*p*_*trend*_*= .600*				*p*_*trend*_*= .436*				*p*_*trend*_*< .001*			

^a^Expected change per ZCTA5 area during 2000–2001.

Supermarkets are the grocery stores of > =50 employees, while small grocery stores are those of <10 employees.

Linear regression models were fitted, adjusted for total population, race/ethnicity composition categories, poverty rate tertiles,% of foreign born population tertiles, urbanization categories, total business size in 2000, the change in business size from 2000 to 2001, region.

W, B, H, M indicate significant difference from the White-, Black-, Hispanic-predominant or Mixed race/ethnicity areas; S, U indicate significant difference from suburban or urban areas; 1, 2, 3 indicate significant difference from the tertile 1, 2, or 3. (p < 0.005 to account for multiple pair-wise comparison).

### Distributional changes in food outlets by local characteristics

**Figure 1:**
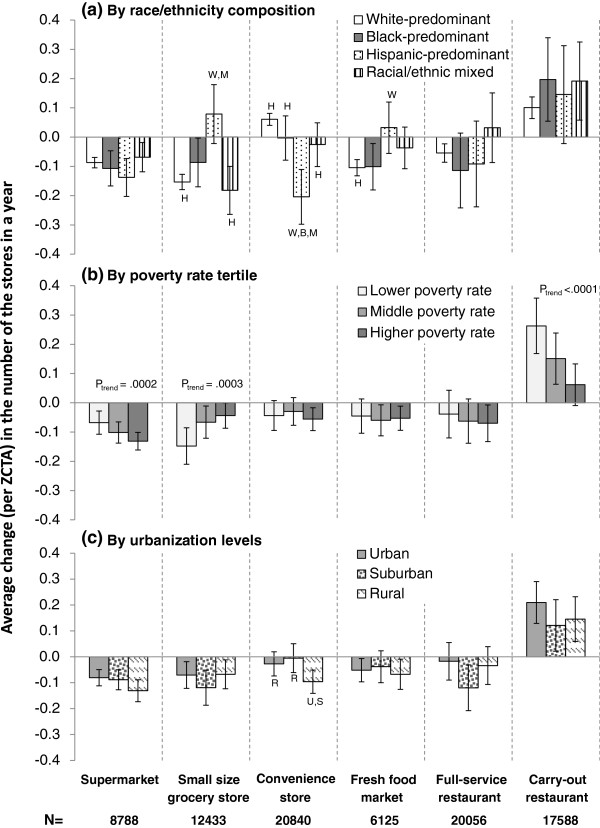
**Changes of food stores/services number in places having the store/service at baseline, by a) race/ethnicity composition, b) poverty rate, c) urbanization level.** 1. Adjusted for local total population, poverty rate,% of foreign born population, urbanization categories, total business size in 2000, the change in business size from 2000 to 2001, and census region. 2. N: number of ZCTA5 areas where the food store of analysis existed in the baseline year. 3. Pairs of characters indicate significant (p < 0.005 to account for multiple pair-wise comparisons) difference from the denoted areas: W, B, H, M indicate different from White-, Black-, Hispanic-predominant and Mixed race/ethnicity areas; R, S, U indicate significant difference from rural, suburban or urban areas. 4. Types of food outlets for the six panels are listed at the bottom of the figure: (from left to right) supermarket, small size grocery store, convenience store, fresh food market, full-service restaurant, carry-out restaurant.

Figure 1 shows areas with the stores/services of interest at baseline and the distributional changes in stores/services across contextual factors after controlling for covariates. For places where there had been any grocery store, the decreased number was significantly larger in mixed racial/ethnic and white-predominant areas than in the Hispanic-predominant areas (p < 0.0001 for both comparisons, Figure 1a). The number of convenience stores declined more in Hispanic-predominant than in the other three areas, if there was any convenience store in 2000. Fresh/specialty food markets grew more in Hispanic-predominant areas than in the other three areas. However, the changes of the food stores/services distribution were not significantly associated with local foreign-born population proportion, so the results were not shown in Figure 1.

In areas with small groceries at baseline, a higher poverty rate was associated with a less decrease in grocery stores (p_trend_ = 0.0003, Figure 1b). This suggests poorer areas may favor the establishments of small-size grocery stores over larger ones. Poorer areas had a greater decrease in supermarkets (p_trend_ = 0.0002) and a lower increase in carry-out restaurants (p_trend_ <0.0001). Urbanization level was only associated with the changed number of convenience stores, which decreased the most in the rural areas (adjusted mean = -0.096, Figure 1c), a significantly greater decrease than urban areas (p = 0.0069) and suburban areas (p = 0.0003).

### Introduction of food outlets by local characteristics

**Figure 2:**
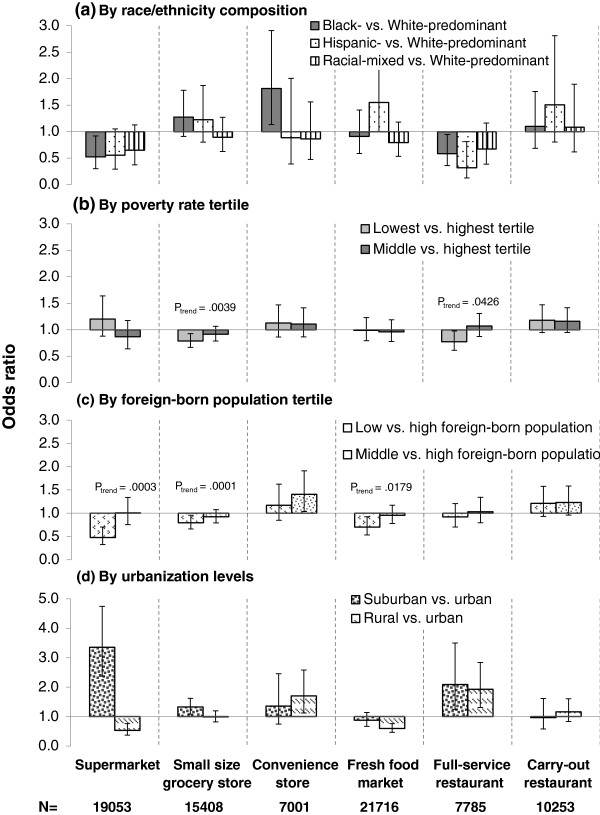
**Odds ratios of 6 types of food stores/services being introduced into places without the stores/services at baseline, by a) race/ethnicity composition, b) poverty rate, c) foreign-born population, d) urbanization level.** 1. Adjusted for local total population, poverty rate, % of foreign born population, urbanization categories, total business size in 2000, the change in business size from 2000 to 2001, and census region. 2. N: number of ZCTA5 areas where the food store of analysis was absent in the baseline year. 3. Types of food outlets for the six panels are listed at the bottom of the figure: (from left to right) supermarket, small size grocery store, convenience store, fresh food market, full-service restaurant, carry-out restaurant.

Among places without supermarkets at baseline, supermarkets were less likely to open in black-predominant than white-predominant areas (OR = 0.52, 95% CI = 0.30-0.92) (Figure 2). Compared to urban areas, supermarkets were more likely to be introduced to suburban areas (OR = 3.35, CI = 2.37-4.74) and less likely to rural areas (OR = 0.53, 0.37-0.76). Meanwhile, the odds of introduction of supermarket into areas with a low foreign-born population was lower than into areas with a high foreign-born population proportion (OR = 0.48, CI = 0.33-0.70).

Small-size grocery stores were more likely to be introduced into suburban than urban areas (OR = 1.32, CI = 1.08-1.62), less likely into areas with fewer foreign-born residents (OR = 0.79, CI = 0.66-0.95), and less likely into areas of lower vs. higher poverty rate (OR = 0.79, CI = 0.67-0.93). Convenience stores were more likely to be introduced into black-predominant than white-predominant areas (OR = 1.82, CI = 1.13-2.91), into areas of middle than high proportion of foreign-born population (OR = 1.41, CI = 1.04-1.91), and into rural than urban areas (OR = 1.70, CI = 1.12-2.58).

New fresh food markets were significantly more likely to enter Hispanic-predominant than white-predominant areas (OR = 1.55, CI = 1.004-2.40), less likely to enter areas of low foreign-born population than areas of high foreign-born population (OR = 0.70, CI = 0.53-0.92), and less likely to be introduced into rural than to urban areas (OR = 0.59, CI = 0.46-0.76). Full-service restaurants were less likely to be introduced into black-predominant (OR = 0.58, CI = 0.36-0.95) and Hispanic-predominant (OR = 0.32, CI = 0.12-0.81) than into white-predominant areas. Introduction of full-service restaurants was significantly higher among suburban/rural than urban areas.

## Discussion

We studied the 1-year changes in food outlet distributions between 2000 and 2001, indicated by six types of food stores and services in the U.S. based on national data. To our knowledge, this is the first of such a study at the national scale. We found several interesting patterns. Local contextual factors such as urbanization level, racial composition and poverty rate were associated with the changes in food outlet distributions. Small-size grocery stores and fresh/specialty food markets increased the most in the Hispanic-predominant areas. Poverty rate was associated with a greater decrease in supermarkets but a greater increase in small-size grocery stores and with slower growth of carry-out restaurants. These contextual factors were associated with the introduction of food stores into places that originally lacked of those stores. These findings demonstrated the heterogeneous patterns of evolving food landscape in the U.S.

People with lower income usually had worse dietary quality than those with higher income [24,25]. Previous research suggests that the availability of supermarkets or larger size food stores in the neighborhood were associated with the residents’ better health and diets [6,15,23]. In our study, neighborhoods’ higher poverty rate was associated with a greater decrease of supermarkets (larger-size grocery stores) and with a lower decrease in smaller grocery stores. This suggests that the population in poorer areas experienced a decreased size in grocery stores during 2000–2001. Compared to larger size food stores, smaller ones may stock more food items of a longer shelf-life (compared to fresh foods) to reduce the loss of profit through food spoilage [19]. It needs further study to understand how size changing of food stores would affect the neighborhood residents’ diet quality.

Hispanic-predominant areas showed a very different picture to areas of other racial/ethnic composition. At baseline, small-size grocery stores, fresh/specialty food markets and convenience stores were the most prevalent in Hispanic-predominant areas. Quantities of small-size grocery stores and fresh/specialty food markets in Hispanic-predominant areas grew faster than areas of other racial/ethnic composition. Moreover, among areas without fresh/specialty food markets, these stores were more likely to be introduced into areas of denser foreign-born population than those areas of lower foreign-born population. Literature shows that foreign-born Hispanic adults and children had more fruits/vegetables or fiber intakes than those born in the U.S., [26,27] which may explain the association of Hispanic and foreign-born population density with the increase of fresh/specialty food markets that sell wholesome foods. Despite the greater consumption of fruits/vegetables, the Hispanics also consumed more “solid fats, alcoholic beverages, and added sugar” than the other race/ethnicity groups [24]. It is unknown whether the more increase of small grocery stores in Hispanic-predominant areas were associated with this. Nevertheless, these phenomena suggest the potential influence of population’s demographic composition on local food outlets dynamics in the U.S., but the pathway between demographic composition and food outlet distributions needs future research to delineate.

Epidemiologic studies have shown that the availability fast food stores might be related to the prevalence of obesity [4,5,23,28-30]. The present study shows a ubiquitous increase in the availability of carry-out and full-service restaurants. In the U.S., the average frequency of dining out has been increasing in the past decades [31,32], i.e. a rising dependence on foods prepared by food outlets. Although it is difficult to label food stores themselves as healthy or unhealthy, the core issue is the foods sold in these stores. If various food stores/services can provide healthier and affordable choices, the environmental obesogenicity could be reduced even though people eat away from home frequently. The calorie-labeling and trans fat ban in New York City are examples of such efforts [33]. There are other attempts to improve the contents of carry-out restaurants and corner stores in places that lack healthier food choices in order to improve the local food environment [34,35]. In addition to food outlets, the penetration of energy-dense foods and drinks into non-food businesses is another attention-catching issue [36]. More studies are needed to understand the influence of an improved selection of food items in stores on customers’ food consumption and health.

The main strength of this study is that we examined the longitudinal changes over time in the quantities of various types of food stores/services throughout the U.S. This helps shed light on factors that may affect the food environment dynamics in the U.S. We looked at a short-term change to prevent the mismatch between Zip code and ZCTA5 over time, and this brought another strength of this study. During the 2000–2001, the issue of the health impact of food environments was not as visible as it has become in the recent years. Thus, the observed difference in food environmental changes related to contextual factors was less “contaminated” by the interventions that began after the food environmental issues caught more of the public’s attention due to increasing concern about the growing obesity epidemic. Meanwhile, our large-scale examination revealed the systematic patterns in food environmental dynamics related to contextual factors in the U.S.

As an initial study looking at the associations between local contextual factors and food stores/services dynamics, some limitations of this study should be noted. First, the changes were studied over a short period between 2000 and 2001. We did not study the change for a longer period in order to prevent mismatch between Census ZCTA5 codes and the Zip codes in business pattern data. The ZCTA5 was designed to coincide with the postal Zip code boundaries in 2000, while ZBP used the postal Zip code, which could be re-designated according to the postal services volume [37]. In addition, average number of all types of establishments in ZBP data decreased from 2001 to 2002, which may reflect the national economic recession in that period. Hence, we did not extend the time frame of this study beyond 2001. Second, only the numbers of stores/services of interest were available, but not the detailed contents such as foods sold in the stores, prices, store facilities, sales volumes and locations. However, our study provides important insight into the changing availability of the food outlets by areas’ different contextual characteristics in the U.S. Third, the data in ZBP may have classification error, but it could be non-differential with respect to the local contextual factors of interest in our study. Some studies suggested commercial database of food establishments as a valid source of data for geographical area equal or larger than census tract level [38,39]. Nevertheless, how the error in ZBP data could systematically vary with local characteristics still need further investigation.

## Conclusion

In conclusion, in the U.S., there are significant changes in local environment at Zip-code area level within a year. Local contextual factors such as racial/ethnic composition, poverty rate and urbanization level would affect local food environment changes. We identified Zip-code area characteristics that may attract or maintain different types of food stores/services. For instance, Hispanic and foreign-born population seemed to attract more food outlets that could sell fresher foods, i.e. grocery stores and fresh/specialty food markets. The universally increasing quantities of carry-out restaurants across areas indicated that they are potential targets for local food environment improvement. These area characteristics may identify communities’ preferences to different types of food providers. Tailored approaches on improving the food contents sold by the locally preferred food stores/services could be an effective way to transform undesirable food environments.

## Competing interests

The authors declare no competing interest.

## Authors’ contributions

H.-J. C. designed research, analyzed data, and wrote the paper. Y. W. guided study design, participated in manuscript preparation and secured related funding. Both authors had primary responsibility for the final content and have read and approved the final manuscript.

## Pre-publication history

The pre-publication history for this paper can be accessed here:

http://www.biomedcentral.com/1471-2458/14/42/prepub
